# The Effect of Body Perception and Marital Adjustment on Cosmetic Surgery Acceptance in Married Women

**DOI:** 10.1007/s00266-025-04888-8

**Published:** 2025-05-14

**Authors:** Esra Ünal, Simge Öztürk, Hatice Aslıhan Hacımuhittinoğulları

**Affiliations:** 1 Department of Midwifery, Faculty of Health Sciences, Osmaniye Korkut Ata University, Osmaniye, 80000 Turkey; 2 Department of Nursing, Faculty of Health Sciences, Bartin University, Bartin, Turkey

**Keywords:** Women, Body perception, Marital adjustment, Cosmetic surgery

## Abstract

**Obejective:**

This study aimed to determine the effect of body perception and marital adjustment on cosmetic surgery acceptance in married women.

**Methods:**

The cross-sectional and correlational study was conducted with 707 married women between July and November 2023. Participant Information Form, Body Perception Scale, Marital Adjustment Test, and Acceptance of Cosmetic Surgery Scale were used to collect the data.

**Results:**

It was determined that the model created to determine the effect of body perception and marital adjustment on cosmetic surgery acceptance in married women was compatible, and the fit indices of the model were within the desired limits. It was determined that body perception and marital adjustment affected cosmetic surgery acceptance, and women with high marital adjustment and low body satisfaction had more positive attitudes toward cosmetic surgery.

**Conclusion:**

Body perception satisfaction affects marital adjustment. Body perception and marital adjustment affect cosmetic surgery acceptance. Longitudinal studies on factors influencing cosmetic surgery acceptance are advised.

**No Level Assigned:**

This journal requires that authors assign a level of evidence to each submission to which Evidence-Based Medicine rankings are applicable. This excludes Review Articles, Book Reviews, and manuscripts that concern Basic Science, Animal Studies, Cadaver Studies, and Experimental Studies. For a full description of these Evidence-Based Medicine ratings, please refer to the Table of Contents or the online Instructions to Authors www.springer.com/00266.

## Introduction

Throughout history, humans have constantly sought beauty and tried to correct their imperfections. This natural need has triggered the search for beauty and elegance and led individuals to cosmetic surgery. The earliest records of such procedures date back to ancient Egypt [1]. The evolution of cosmetic surgery, especially in America, has accelerated with the increasing prosperity and individualism of the modern world. Developing technology has contributed to the spread of cosmetic surgery by reducing pain and infection risk in surgical operations. In addition to these developments, cultural changes and the rise of mass beauty ideals have affected women more [2].

The latest global research report released by the International Society of Aesthetic Plastic Surgery (ISAPS) in 2023 indicates an essential surge in aesthetic procedures throughout the past four years. The findings indicated a rise of 41.3% in surgical aesthetic treatments and 57.8% in non-surgical aesthetic procedures. According to ISAPS, Turkey ranks fourth in cosmetic surgery operation rates, with 470,875 surgical events in 2022 [3]. An estimated 85.7% of aesthetic treatments are conducted on women, although there has been a notable 18% rise in procedures performed on men [3, 4].

The potential side effects of cosmetic surgery encompass discomfort, nausea and vomiting, bleeding, cardiac complications, nerve damage, and infection [5, 6]. Furthermore, cosmetic surgery carries significant risks, including edema, scleral appearance, hypersensitivity resulting from increased use of synthetic facial injections, cutaneous vascular hazards leading to skin and tissue death, blindness, and cerebral embolism [7]. While some research in the literature proposes that cosmetic surgery may provide psychosocial advantages [8, 9], other studies indicate that this situation directly contributes to the emergence of mental health issues following aesthetic surgery [10, 11]. Worldwide research suggests that women have lower levels of satisfaction with their physical appearance and are more inclined to choose cosmetic surgery to enhance their look [12, 13]. An analysis of the factors contributing to women’s decision to undergo cosmetic surgery operations includes body dissatisfaction, the media’s portrayal of women in an idealized manner, low self-esteem, and observing positive cosmetic surgery experiences of others [14]. Research indicates that the negative emotions and beliefs individuals have about their bodies have a substantial impact on both their sexual satisfaction and the overall harmony within their marriages [15, 16].

The decision of married women to seek cosmetic surgery to enhance their physical appearance underscores the significance of physical attractiveness as one of the many elements influencing marital satisfaction [17]. Research undertaken to assess the impact of cosmetic surgery on marital satisfaction among married women found that the anticipation of a favorable outcome after the procedure was a significant determinant that enhanced marital satisfaction in women who had cosmetic surgery [17].

Determining the reasons for resorting to cosmetic surgery may contribute to the creation of cause-oriented approaches before women resort to the dangers and financial expenses brought by surgical operations. In the literature, there are studies examining body image [18], body dissatisfaction in young women [19], and the effect of cosmetic surgery on sexual self-esteem in married women [1], but there is no study examining the effect of body perception and marital adjustment on cosmetic surgery acceptance in married women who do not undergo cosmetic surgery. This study aimed to determine the effect of women’s body perception and marital adjustment on cosmetic surgery acceptance.

## Method

The cross-sectional and correlational study was conducted with 707 married women between July 2023 and November 2023. In this study, we tried to determine the effect of body perception and marital adjustment on cosmetic surgery acceptance in married women.

### Population and Sample of the Study

The research population comprises married women residing in Turkey. The minimum number of married women to be included in the study sample was calculated as 384 women using the unknown population formula (*n* = *t* 2 – *p* − *q/d* 2) (*d* = 0.05), *t* = 1.96, *P* = 0.5, *q* = 0.5). The study included 707 married women. Based on the post hoc power analysis conducted on the data collected from 707 participants, the effect size was determined to be 0.164. The study’s power was 99% at a 95% confidence level.

### Inclusion Criteria

Women who were married, had internet access, had not undergone plastic surgery before, and agreed to participate in the study were included.

### Exclusion Criteria

Women in pregnancy and postpartum (first 1 year) were excluded from the study.

### Removal Criteria

Participants who incompletely completed the online questionnaire were excluded from the study.

### Data Collection Tools

Data were collected using the Participant Information Form, Body Perception Scale, Marital Adjustment Scale, and Cosmetic Surgery Acceptance Scale.

### Participant Information Form

The Participant Information Form, which includes questions about age, employment status, age and employment status of the spouse, year of marriage, social media use, social media accounts used, and plastic surgery, includes a total of 13 questions.

### Body Perception Scale

A Turkish adaptation of the Body Perception Scale (BCS), first created by Secord and Jourard in 1953, was produced by S. Hovardaoğlu in 1989 [20, 21]. The Cronbach’s alpha internal consistency coefficient of the scale was calculated by Hovardaoğlu to be 0.91. The scale comprises 40 items that are responded to using a five-point Likert scale. Every individual item delineates an organ or anatomical structure (such as an arm, leg, or face) or a physiological role (such as the degree of sexual activity). Ratings are assigned on a scale of “I strongly dislike it—I strongly dislike it—I am undecided—I like it—I like it very much.” Each item on the scale is assigned a score ranging from 1 to 5. Higher scores on the scale suggest lower levels of satisfaction with body parts or functions. The present investigation revealed a Cronbach’s alpha coefficient of 0.947 for the BCS.

### Marital Adjustment Test

The Marital Adjustment Test (MAT), originally created by Locke and Wallace in 1959 and subsequently translated into Turkish by Kışlak in 1996, has undergone validity and reliability investigations [22, 23]. Its purpose is to assess levels of satisfaction and adjustment within marital partnerships. This scale comprises 15 items with varying numbers of alternatives, resulting in a total score ranging from 0 to 60. Based on this scale, individuals who get a score of 43 points or higher are deemed compatible in terms of marital relationships, while those who score below this threshold are regarded incompatible. The reliability of the scale was assessed by Kışlak (1996) by the computation of Cronbach’s alpha coefficient and two-half reliability coefficient. The Cronbach’s alpha measurement of internal consistency was determined to be 0.80, while the two-half reliability coefficient was found to be 0.67 [23]. The present investigation revealed a Cronbach’s alpha coefficient of 0.849 for the scale.

### Acceptance of Cosmetic Surgery Scale

In 2017, Karaca et al. did an assessment of the Turkish validity and reliability of the scale created by Henderson-King and Henderson-King in 2005 [24, 25]. The measurement tool consists of 15 components divided into 3 sub-dimensions. The scale is divided into three sub-dimensions: personal, societal, and thoughts. A reverse coding is applied to the 10 th item on the scale. The assessment depends on both the three sub-dimensions and the overall score of the scale. The ACSS score distribution spans from 15 to 105. Elevated scores in the sub-dimensions and the overall score of the scale suggest that there is a favorable orientation toward cosmetic surgery. The findings of Henderson-King’s study indicated a good level of internal consistency for the scale, with a Cronbach alpha ranging from.91 to.93. A stated Cronbach alpha coefficient of 0.92 was obtained for the Turkish version. A Cronbach’s alpha coefficient of 0.966 was obtained for the scale in this investigation.

### Data Collection Process

In the process of collecting the data for the research, a questionnaire form created using the online Google Forms application. The link to this questionnaire form was sent via online social media platforms (WhatsApp, Instagram) and e-mail. The purpose of the study and inclusion criteria were explained at the beginning of the questionnaire form. No personal data were requested during the data collection process. The questionnaire form was designed so that each participant could fill it out once to ensure data integrity and security.

### Data Analysis

Statistical software packages IBM SPSS 25 and AMOS 22 were utilized for data analysis. Skewness and kurtosis values were employed to assess the normality of the data distribution. An investigation was conducted using structural equation modeling to examine the correlation between body perception, marital adjustment, and acceptance of cosmetic surgery. Fit indices (*χ*^2^/standard deviation, goodness of fit index (GFI), adjusted goodness of fit index, comparative fit index (CFI), standardized root mean square residual, and root mean square error of approximation (RMSEA)) were used to evaluate model fit. An acceptable statistical significance level was set at *p* < 0.05.

### Ethical Statement

Approval was obtained from the Ethics Committee of a state university to conduct the research. In the online link created for the research, participants were informed about the purpose, and method of the research and it was explained that participation was voluntary and would not cause any harm. The study duly secured the agreement of the subjects and strictly followed the Declaration of Helsinki to safeguard individual rights.

## Results

### Participants

The distribution of socio-demographic and cosmetic surgery acceptance characteristics of the 707 women who participated in the study is shown in Table 1. Of the women participating in the study, 59% were between the ages of 18–35, 59.5% were not working, 47.2% had husbands between the ages of 18–35, 85.9% had husbands who were working, 59.1% had income equal to expenses, 78.8% were between the ages of 14–25, 56% had been married for 1–10 years, 92.2% use social media, 52.2% do not use a shop/filter before sharing after taking a photo, 50.5% do not have a person who has undergone aesthetic procedures in their immediate environment, 77.5% do not think about having aesthetic procedures, and the reason for 27.6% not having aesthetic procedures was determined as religious reasons.

**Table 1:** Distribution of socio-demographic and cosmetic surgery acceptance characteristics of women

			ACSS		
	*n*	%	Median(Min-Max)	Test value	*p* value
Age					
18–35 years	417	59	49 (15–105)^c^	58.783^a^	***p*** < **0.001**
36–43 years	123	17.4	41 (15–105)^d^		
44 and above	167	23.6	31.5 (15–105)^e^		
Employment status					
Working	286	40.5	50 (15–105)	45180.000^b^	***p*** < **0.001**
Not working	421	59.5	36 (15–105)		
Age of Spouse					
18–35 years	334	47.2	48 (15–105)^c^	48.274^a^	***p*** < **0.001**
36–43 years	134	19	47 (15–105)^cd^		
44 and above	239	33.8	33 (15–105)^e^		
Spouse’s employment status					
Working	607	85.9	45 (15–105)	24655.000	**0.003**
Not working	100	14.1	33 (15–105)		
Income status					
Income less than expenditure	168	23.8	36 (15–105)	0.704^a^	0.703
Income equal to expenditure	418	59.1	44.5 (15–105)		
Income more than expenditure	121	17.1	46 (15–105)		
Marriage age					
14–25 years old	557	78.8	41 (15–105)	39696.500	0.349
26–43 years	150	21.2	46 (15–105)		
Duration of marriage					
1–10 years	396	56	48.5 (15–105)	43294.500	***p*** < **0.001**
11 and more years	311	44	34 (15–105)		
Social media use status					
Yes	652	92.2	45 (15–105)	8639.000	***p*** < **0.001**
No	55	7.8	21 (15–105)		
WhatsApp					
Yes	627	88.7	54 (15–105)	16779.000	***p*** < **0.001**
No	80	11.3	34 (15–105)		
Instagram					
Yes	541	76.5	59 (15–105)	29769.500	***p*** < **0.001**
No	166	23.5	33.5 (15–105)		
Facebook					
Yes	201	28.4	48 (15–105)	47677.500	0.195
No	506	71.6	52.5 (15–105)		
TikTok					
Yes	128	18.1	53 (15–105)	35100.000	0.349
No	579	81.9	51 (15–105)		
YouTube					
Yes	449	63.5	54 (15–105)	51054.000	**0.009**
No	258	36.5	47 (15–105)		
Twitter					
Yes	159	22.5	63 (15–105)	36617.500	**0.002**
No	548	77.5	48 (15–105)		
LinkedIn					
Yes	39	5.5	71 (25–104)	10362.500	**0.032**
No	668	94.5	50 (15–105)		
Other					
Yes	80	11.3	32.5 (15–105)	17277.500	***p*** < **0.001**
No	627	88.7	54 (15–105)		
Use of shop/filters before sharing after taking a photo					
Yes	190	26.9	58 (15–105)^c^	85.766^a^	***p*** <** 0.001**
No	369	52.2	41 (15–105)^d^		
I do not share	148	20.9	33 (15–100)^e^		
Has anyone around you undergone aesthetic surgery?					
Yes	350	49.5	55 (15–105)	37804.500	***p*** < **0.001**
No	357	50.5	34 (15–105)		
Are you considering plastic surgery?					
Yes	159	22.5	48 (34–79)	15079.000	***p*** < **0.001**
No	548	77.5	42 (15–105)		
The reason why you don’t want to have it done					
Economic reasons	64	9.1	93 (23–105)	168.956^a^	***p*** < **0.001**
Fear of surgical procedures	50	7.1	61 (26–105)		
The thought that it won’t be what I want	29	4.1	54 (16–105)		
Religious reasons	195	27.6	28 (15–99)		
My family’s perspective on plastic surgery	23	3.3	46 (21–98)		
Negative attitude of those who have undergone plastic surgery in my environment/media	25	3.5	50 (24–101)		
Other	162	22.9	41 (15–105)		

Bold values indicate statistical significance (*p* < 0.05)

^a^Kruskall–Wallis test

^b^Mann–Whitney U test

^c–e^There is no difference between group with the same letter

The mean scores of the women in the study were 84.26 ± 28.47 on the Body Perception Scale, 45.29 ± 9.07 on the Marital Adjustment Scale, and 55.85 ± 29.32 on the Cosmetic Surgery Acceptance Scale. There was a negative and moderately significant correlation between the Body Perception Scale and the Marital Adjustment Scale (*r* = −0.327, *p* < 0.001). There was a significant positive correlation between the Marital Adjustment Scale and the Cosmetic Surgery Acceptance Scale (*r* = 0.110, *p* < 0.001) (Table 2).

**Table 2:** Correlation table

	x̄ ± SD	M (Min-Max)	1	2	3	4	5	6	7	8
1. Total score of MAT	45.29 ± 9.07	47 (11–59)	1							
2.Agreement	36.64 ± 7.68	38 (3–46)	0.984^**^	1						
3. Style	8.65 ± 2.03	9 (2–13)	0.740^**^	0.608^**^	1					
4. Total score of ACSS	55.85 ± 29.32	52 (15–105)	0.110^**^	0.108^**^	0.084^*^	1				
5. Interpersonal	21.36 ± 11.12	23 (5–35)	0.085^*^	0.078^*^	0.085^*^	0.918^**^	1			
6. Social	15.15 ± 9.99	11 (5–35)	0.106^**^	0.107^**^	00.067	0.917^**^	0.735^**^	1		
7. Consider	19.34 ± 10.52	17 (5–35)	0.117^**^	0.116^**^	0.081^*^	0.946^**^	0.803^**^	0.828^**^	1	
8. Total score of BCS	84.26 ± 28.47	83 (40–190)	− 0.327^**^	− 0.327^**^	− 0.225^**^	0.053	0.043	0.060	0.046	1

*MAT* marital adjustment test. *ACSS* acceptance of cosmetic surgery scale. *BCS* body-cathexis scale. *x̄* mean. *SD* standard deviation. *M.n* minimum value. *Max* maximum value

*p < 0.05

**p < 0.001

### Path Analysis

**Fig. 1:**
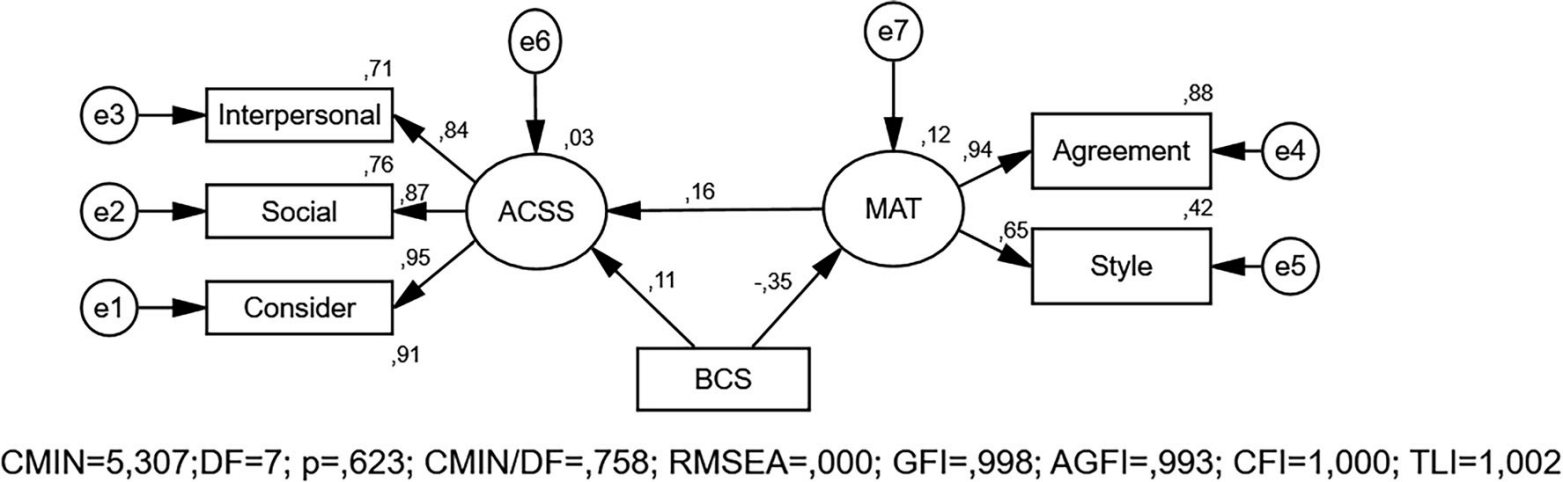
Path analysis of factors affecting cosmetic surgery acceptance

The path analysis model in the study is presented in Figure 1. After the structural equation model was established, CMIN = 5.307, DF = 7, CMIN/DF = 0.758, RMSEA = 0.000, GFI = 0.998, AGFI = 0.993, CFI = 1.000, TLI = 1.002 and SRMR = 0.0067. It was determined that all of the fit criteria were within acceptable limits (Tables 2, 3).

The path coefficients of the interpersonal, social, and consider sub-dimensions of the ACSS were statistically significant, and the standardized values of these path coefficients were between 0.844 and 0.951 (*p* < 0.001). An increase in the body perception scale leads to a decrease of 0.349 units in the marital adjustment scale score. As satisfaction with body perception decreases, marital adjustment also decreases. An increase in the marital adjustment scale score leads to an increase of 0.165 units in cosmetic surgery acceptance scale score. It was determined that women with high marital adjustment had a more positive attitude toward cosmetic surgery. An increase in the body perception scale leads to an increase of 0.11 units in the cosmetic surgery acceptance scale. It was determined that women exhibited a more positive attitude toward cosmetic surgery as their satisfaction with body perception decreased. This model showed that body perception and marital adjustment are effective in cosmetic surgery acceptance of married women (Table 3).

**Table 3:** Path analysis results

			*β*0	*Β*1	S.E.	C.R.	*P*	*R*^2^
Agreement	←	MAT	0.936	1				0.875
Style	←	MAT	0.65	0.184	0.023	8.137	**< 0.001**	0.413
Interpersonal	←	ACSS	0.844	0.938	0.029	32.463	**< 0.001**	0.712
Social	←	ACSS	0.871	0.869	0.025	34.438	**< 0.001**	0.758
Consider	←	ACSS	0.951	1				0.905
*Structural model*								
MAT	←	BCS	−0.349	−0.088	0.01	−9.18	**< 0.001**	0.122
ACSS	←	MAT	0.165	0.229	0.067	3.411	**< 0.001**	0.027
ACSS	←	BCS	0.11	0.039	0.015	2.631	**0.009**	

Bold values indicate statistical significance (*p* < 0.05)

*MAT* marital adjustment test, *ACSS* acceptance of cosmetic surgery scale, *BCS* body-cathexis scale, *β0* standardized coefficient, *β1* unstandardized coefficient, *SE,* standard error, *C.R.* critical ratio, *R* regression coefficient

## Discussion

With the developments in technology and health, the concept of beauty is also changing. The ideal body image in the media affects women’s understanding of beauty and leads to the desire to achieve idealized bodies [26]. This situation directs women to cosmetic surgery procedures [1]. It is essential to determine the factors affecting the acceptance of cosmetic surgery to provide cause-oriented approaches before surgery.

In this study, it was determined that the acceptance of cosmetic surgery increased among women whose husbands and wives were in the younger age group and who worked. In the study, it was determined that the acceptance rate of cosmetic surgery increased in women who had a marriage duration of less than ten years and who used social media, WhatsApp, Instagram, YouTube, Twitter, and LinkedIn. In addition, it was determined that the acceptance of cosmetic surgery is higher in women who use shop/filter after taking photos, who have undergone aesthetic surgery in their immediate surroundings, and who are considering having aesthetic surgery. In the study conducted by Gesto et al. [26], it was determined that the acceptance of cosmetic surgery was higher in women whose husband and wife were in the young age group and who were working, who had less than ten years of marriage, who used social media, who used WhatsApp, Instagram, YouTube and LinkedIn, who used shop/filtering before sharing after taking photos, who had undergone aesthetic surgery in their immediate surroundings, who were considering having aesthetic surgery, and whose economic means were suitable for plastic surgery [26]. However, in this study, it was determined that seeing photos of celebrities on social media increased body dissatisfaction and increased cosmetic surgery acceptance [26]. In the study conducted by Chen et al. [27], it was determined that body perception decreased as YouTube, WhatsApp, shop usage status, time spent on social media, and photo sharing increased, while cosmetic surgery acceptance increased. In addition, the study, determined that the acceptance of cosmetic surgery increased in individuals who evaluated their value according to their physical appearance [27]. Nerini et al. [26] found that the use of social media changed the body image of women, and the changed body image increased the acceptance of cosmetic surgery. Alkhathami et al. [28] conducted a study with 1685 women and found that 38.9% of women accepted cosmetic surgery at a high level and 61.1% at a low level. This study determined that acceptance of cosmetic surgery increased as education level employment status increased, and income status decreased. It was found that women who had undergone cosmetic surgery before or knew someone who had undergone cosmetic surgery were more likely to accept cosmetic surgery. In this study, it was determined that 29.6% of the participants used Snapchat, Instagram, and TikTok to take selfies and that the use of social media, financial freedom and income increase, dissatisfaction with body appearance, and family and friend pressure will increase the use of cosmetic surgery [28]. In the study conducted by Seo and Kim [29], it was determined that women’s acceptance of cosmetic surgery increased as the stress level, exposure to cosmetic surgery advertisements, cosmetic surgery experience, and cosmetic surgery thinking increased. In addition, it was stated that acceptance of cosmetic surgery increased as depression increased and self-esteem decreased.

In this study, when the reasons for not having cosmetic surgery were analyzed, it was determined that the highest rate (27.6%) was religious reasons, followed by obstacles and fear factors. This result is a striking result for Turkey, which is one of the countries where cosmetic surgery procedures are performed intensively. In a study examining the reasons for women to apply for cosmetic surgery in the literature, it was stated that religiosity was practical on cosmetic surgery and that older women who had not undergone cosmetic surgery before thought that cosmetic/aesthetic surgery was contrary to religious belief and even sinful. In a study examining the reasons for having, and not having cosmetic surgery in the literature, it was stated that the reasons for not having cosmetic surgery were satisfaction with one’s appearance, fear, and obstacles [30]. The research result is similar to the literature.

In this study, it was determined that women with high marital adjustment and low body satisfaction had more positive attitudes toward cosmetic surgery. In a study (2018) evaluating marital adjustment and self-concept after cosmetic surgery in married women, it was stated that the expectation of a positive outcome regarding cosmetic surgery was effective on marital satisfaction and self-concept was an important factor in applying for cosmetic surgery [17]. In a study evaluating the effect of genital plastic surgery on body image and sexuality in couples, it was emphasized that female genital plastic surgery improves women’s body image and sexual function, which leads to a healthier marital relationship [31]. In the study conducted by Esmalian et al. (2020), it was determined that women’s sexual self-esteem, well-being levels, and body image increased as the rate of cosmetic surgery increased. Increased sexual self-esteem and body image positively affect marital adjustment [1, 15, 16].

### Limitations

Since the study was conducted using an online form, those who knew how to use a computer/phone to fill in the form, and knew how to fill in the online form were included in this study. This limited the accessibility of the study to everyone.

### Strengths of the Research

The large sample size, socio-demographically homogeneous (inclusion of women who have not undergone cosmetic surgery before), and the use of previously validated measurement tools constitute the strengths of the study in terms of the reliability and generalizability of our findings.

## Conclusion and Recommendations

In this study, it was determined that the acceptance of cosmetic surgery was higher in women whose husband and wife were young, who were working, who had a higher income, whose marriage period was less than ten years, who used social media (WhatsApp, Instagram, YouTube, Twitter, LinkedIn), who used shop/filter before sharing after taking photos, who had aesthetic operations in their immediate surroundings, and who were considering having aesthetic surgery. It was determined that body perception and marital harmony affected cosmetic surgery acceptance, and women with high marital harmony and low body satisfaction had more positive attitudes toward cosmetic surgery. It is recommended that healthcare professionals should consider marital harmony and body image when evaluating women’s acceptance of cosmetic surgery and question their relationship.

## Data Availability

I confirm the data availability confirmation.
